# Wider health needs in attention deficit hyperactivity disorder from lived and professional experience: a qualitative framework analysis

**DOI:** 10.1136/bmjopen-2023-083539

**Published:** 2024-08-14

**Authors:** John Ward, Audrey McBride, Rebecca Gudka, Kieran Becker, Tamsin Newlove-Delgado, Anna Price

**Affiliations:** 1 University of Exeter Medical School, Exeter, UK; 2 Department of Psychiatry, University of Oxford, Oxford, UK

**Keywords:** qualitative research, psychiatry, primary health care

## Abstract

**Abstract:**

**Objectives:**

This study aimed to explore the perspectives of people with attention deficit hyperactivity disorder (ADHD), their supporters and primary care professionals (PCPs), on the wider physical and mental health needs of people with ADHD and the support currently available.

**Design:**

Qualitative semi-structured interviews, analysed using reflexive thematic analysis.

**Setting:**

Five general practice surgeries across England.

**Participants:**

Participants with lived experience (people with ADHD and their supporters (n=11)) and PCPs (n=9) (eg, general practitioners and practice managers), recruited via clinical academic networks and previous work packages of this study.

**Results:**

We generated three major themes in relation to ADHD, using reflexive thematic analysis: understanding health, barriers to health and addressing health. Within these, participants reflected on mental and physical health challenges, as well as wider social difficulties and variability in support offered/accessed.

**Conclusions:**

This study highlights that health problems in ADHD are complex and rooted both in individual factors (eg, mental health) and social factors (eg, support). This study also highlights the differences in expectations and fulfilment of healthcare.

**STRENGTHS AND LIMITATIONS OF THIS STUDY**

- This study recruited patients from five different general practice surgeries across England.
- We recruited professionals from a range of professional backgrounds into our healthcare professional group.
- Currently, given significant variation in attention deficit hyperactivity disorder (ADHD) care, this study may not be entirely representative of ADHD care nationally in the UK.

## Background

 Attention deficit hyperactivity disorder (ADHD), characterised by inattention, hyperactivity and impulsivity, is the most common neurodevelopmental disorder worldwide, with a global prevalence of 5%–7% in children and adolescents and persistence into adulthood in 60%–70% of people.^1^ Both its commonality and its significant psychological, physical and social sequelae make comprehensive understanding and effective, evidence-based management of ADHD imperative.

In the UK, the recommended structure for ADHD service provision varies by age and factors such as psychiatric comorbidity, but typically medication prescribing occurs through shared care agreements between primary and secondary care services.^2^ This agreement entails the initial diagnosis and treatment of ADHD by a specialist in a secondary care setting (eg, a specialist ADHD service) and the ongoing issuing of prescriptions, monitoring and physical health checks by general practitioners (GPs) in primary care.^3^ Patients should be reviewed annually by a specialist in ADHD. However, many varied regional and local service models exist across the UK.^3 4^ Furthermore, primary care clinicians express a lack of confidence in managing ADHD and the complex comorbidities associated with it.^2 3^

Comorbidity and mortality rates are high in people with ADHD, and they have both a greater risk of certain health conditions compared with the general population and a greater exposure to a range of health risks. Research has demonstrated elevated comorbidity of ADHD and obesity, asthma, atopy, diabetes mellitus, hypertension, psoriasis, epilepsy, sexually transmitted infections, ocular abnormalities, immune disorders, metabolic disorders and lung and cardiovascular disease.^5^,^12^ Research has also evidenced increased exposure to physical health risks among people with ADHD. These include substance use disorders, accidental injuries, teenage pregnancy, suicide and poor quality of life.^57 13^,^16^ Enhanced integration of ADHD management at a primary care level has been recommended to provide the holistic approach that these comorbidities and risks require.^17^

In developing a care model that emphasises the role of primary care, it is important to answer outstanding questions about how people with ADHD conceptualise their broader health needs (ie, beyond the management of ADHD itself) and what support they currently receive. Likewise, it is important to understand the perspectives of primary care professionals on these issues. The views of people with lived experience and other key stakeholders can then shape research priorities and help to improve care and interventions. Our previous scoping review on psychosocial interventions highlighted the need for a fuller conceptualisation of health and care needs in ADHD, as well as an increased availability and evaluation of interventions (Ward *et al*, under review). This study aimed to explore the perspectives of people with ADHD, their supporters and primary care professionals on the wider physical and mental health needs of people with ADHD and the support currently available.

## Methods

This study forms part of the second work package of the National Institute for Health Research-funded Mapping ADHD services in Primary Care (MAP) study.^18^ This study received ethics approval from the Health Research Authority (Yorkshire and the Humber—Bradford Leeds Research Ethics Committee, reference 22/YH/0132).

### Sampling and recruitment

The study recruited from two broad groups: primary care professionals (GPs, practice managers (PMs) and allied healthcare professionals) and people with lived experience of ADHD (people aged 16 and over with ADHD and their supporters/carers).

All participants had to live in England to participate in the study. Furthermore, all participants with personal experience had to either be an adult (over 16 years old) with ADHD or directly support an adult with ADHD. Professional participants had to work in a primary care setting in either patient-facing or non-patient-facing roles. There were no explicit exclusion criteria if the inclusion criteria were satisfied. Participants were identified through contact with five GP practices located in different integrated care systems (ICSs) across England. Practices were selected through purposive sampling to cover a range of experiences and increase information power (eg, urban vs rural locations, university populations and deprived vs affluent populations). Four of these practices were urban, and one was rural. Three of these practices were in areas of high deprivation, and one of these practices served a large student population. These GP surgeries were identified primarily from the survey run as part of the work package of one of the MAP studies,^18^ where respondents had agreed to be contacted regarding further research. Practices were also identified through the South West Clinical Research Network and through academic contacts and research partners, such as the UK Adult ADHD Network. Participating practices agreed to disseminate information about the study to eligible staff and patients, who were asked to contact the research team if they were interested in taking part. Supporters (eg, parents/friends) were recruited via the people with ADHD who participated in the study.

In total, 20 participants were recruited, consisting of five GPs, five PMs, one mental health worker (MHW), six people with ADHD (P) and three supporters of people with ADHD (S). The demographics of the participants are given in table 1.

**Table 1:** Participants and demographics (adapted from Gudka *et al*)^49^

Participant	Number of participants	Gender (M:F)	Ethnicity	Additional information
Lived experience people with attention deficit hyperactivity disorder	6	2 Male4 Female	6 White	
Lived experience supporters	3	1 Male2 Female	3 White	
Primary care professionals	11	3 Male8 Female	8 White2 Asian1 Mixed	5 General practitioners5 Practice managers1 Well-being worker

### Interview methodology

Interviews were conducted virtually (JW, RG, KB, TND and AP), given the geographic spread of this study. Interviews took place between March and June 2023. They were either conducted securely through Microsoft Teams (MS Teams) or by mobile phone, depending on participant preference. Participants’ electronic written consent was taken via email prior to interviews and reconfirmed at the time of the interview. Participants were compensated for their time with a £25 voucher.

RG, TND and AP already have qualitative interview experience. JW and KB therefore received training on the topic guide and qualitative interviewing from AP and RG. The professional backgrounds of the interviewers were clinical academics (JW and TND), student researchers (RG and KB) and research fellows from a psychology background (AP).

Interviews were semi-structured and approximately 1 hour in length, and each participant only participated in one interview. Apart from one interview with two professional interviewees, all participants partook in one-to-one interviews with a researcher.

Interviews were recorded, transcribed verbatim by a trusted service and deleted. Participants did not see transcripts post interview. Interviews were up to 1 hour long and based on predetermined topic guides. These topic guides reflected the study aims and were developed in conjunction with the study research advisory groups (RAGs), ensuring patient and public involvement (PPI). The topic guides were also piloted within RAGs. Two versions were created, with one tailored towards primary care professionals and the other tailored for those with lived experiences (ie, people with ADHD and their supporters). The topic guides covered five key areas: how people with ADHD access primary care, healthcare support in primary care (including pharmacological and non-pharmacological support), healthcare and support for wider healthcare needs through primary care, information and resources and digital solutions to help manage ADHD and final reflections. The questions relating to this paper are given in table 2, while the full topic guide is available in online supplemental file 1.

**Table 2:** Topic guide in relation to physical health

Topic guide for participants with lived experience	Topic guide for primary care professionals
Healthcare and support for wider health needs through primary care.This section asks about your experiences of healthcare and support from your general practitioner for wider physical and mental health needs (when you have ADHD). Do you (or does the person your support) have any co-existing health challenges? (mental health/neurodevelopmental difference/physical). Have you had a chance to discuss your health more widely with your primary care provider? (mental health/neurodevelopmental difference/physical). Are you aware of any health risks faced by people with ADHD? (Have you had a chance to discuss this or received advice? Awareness resubstance use, smoking and other risk-taking behaviours; any signposting to support; harm reduction strategies, etc). What do you feel your practice could do to help you to get your wider health needs met when you have ADHD? Do you have anything to add?	Providing care and support for wider mental and physical health through primary care to people with ADHD.This section asks about your experiences of providing for the wider neurodevelopmental, mental and physical healthcare needs of young people and adults with ADHD. Is there anyone at your practice or in the PCN with a special interest in mental health or neurodevelopmental difference that can provide psychological or social support to someone with ADHD? Please tell us about any additional roles that are in place at your practice or in the PCN that could provide support to a patient with ADHD (social prescriber, pharmacist,mental health worker… What are your experiences in relation to this?). Are there any things you consider when providing for the wider healthcare needs of patients with ADHD? (neurodevelopmental difference/wider mental and physical health). What do you think are the most important increased health risks associated with having ADHD? (How relate to ADHD?) Do you or staff at your practice give targeted advice/support on any of these as part of your approach? If so, please describe. Do you have anything to add?

ADHDattention deficit hyperactivity disorderPCNprimary care network

### Research advisory group

There were two RAGs for this study: a healthcare professional group (n=7) and a lived experience group (n=8). Our healthcare professional group consisted of an ADHD specialist nurse, a pharmacist, a social prescriber, an occupational therapist and three GPs. Our lived experience group was comprised of five young people aged between 16 and 25, from diverse ethnic backgrounds across England, and three parents of young people with ADHD. None of the RAG participants took part in the study.

Four meetings for each group were held at approximately 6 monthly intervals throughout the project. Following RAG involvement, where we consulted on interview topic guides, it was noted that our lived experience participants were very encouraging of our questions regarding health and ADHD.

Early qualitative findings from this project were shared with the RAGs, in line with the plans for the further development of this work.

### Qualitative analysis

For this project, we used reflexive thematic analysis, using framework methods in the manner described in Byrne and taking a critical realist approach.^19 20^ This was undertaken between July and August 2023. For details, see the MAP study protocol.^18^

A preliminary framework was developed using an inductive approach to the data and assessing information power as the analysis progressed.^21^ This involved line-by-line coding of initial transcripts by researchers (AP, RG, JW and KB) and code refinement of all transcripts. Data collated under codes relating to perceptions of broader health and healthcare needs in ADHD were identified and reviewed, and a framework matrix was created. Data coded under other codes were reviewed to ensure no relevant data had been missed. Researchers then produced column summaries, which were used to generate themes and subthemes relating to this research question.

### Reflexive statement

The professional backgrounds of the interviewers were clinical academics (JW and TND), student researchers (RG and KB) and research fellows from psychology backgrounds (AP). With respect to previous and lived experience, AP is the parent of a young person with ADHD, which, combined with her involvement in 92 interviews with young people with ADHD and their supporters (in her role as a researcher), has shaped her approach. She also works closely with and has been involved in 52 interviews with healthcare professionals supporting people with ADHD in secondary care settings. Throughout the study, AP held regular meetings with the team to facilitate reflections on underlying assumptions and presuppositions about the sample and topic area. Reflective journals were kept to aid the process of coding and theme generation by highlighting and separating the personal engagement of researchers with the study and its findings (eg, clinical academics dealing with criticism of clinical services).

### Patient and public involvement

PPI was used in the development of the original MAP study protocol,^18^ with the research idea developed in partnership with people with ADHD and their supporters and originating from lived experience perspectives, as well as in the development of the topic guide for this qualitative study. This paper and its findings have been disseminated to PPI advisors.

## Results

From the 19 interviews conducted with 20 participants, three major themes were generated. These have been tabulated in table 3.

**Table 3:** Themes and subthemes generated in reflexive thematic analysis

Theme	Subtheme	Theme description
Understanding health in ADHD	Identified health risks	This theme denotes participants’ discussions of their health problems and how they may relate to ADHD.
	Conceptualisation of ADHD in health	
Barriers to health in ADHD	Behavioural risks	This theme denotes barriers to health, both in terms of things that may be impacting directly on the health of people with ADHD (eg, risky behaviour) or indirectly (eg, difficulty accessing appropriate healthcare).
	Inadequate support	
Addressing health in ADHD	Empowerment and enablement in ADHD	This theme denotes participants’ views on how better incorporated and holistic management of ADHD could be achieved, or reflections on pre-existing practices.
	Specific recommendations	

ADHDattention deficit hyperactivity disorder

### Understanding health in ADHD

In this theme, participants discussed their perceptions of broader health problems and how they saw them relating to ADHD.

### Identified health risks

Both professional and lived experience groups reflected a variety of physical and mental health challenges experienced by people with ADHD.

Yes, I have fibromyalgia. And then I also have post-traumatic stress disorder (PTSD), depression, anxiety and suspected autism. But again, I can’t get diagnosed with that at the moment because the waitlist is a really long time. (Person with ADHD)

He (GP’s child) just did not eat on methylphenidate. It kept him in mainstream school, but he did not eat and he did not particularly sleep. So, he has no cut-off. He will eat for Britain. Yeah. So, I think he is a little bit on the overweight side. (GP)

Some participants felt there were additional challenges that exacerbated physical and mental health problems related to ADHD, for example, low self-esteem and self-neglect.

Oh! I think mental health problems. I think if you are constantly told when you are little that you are naughty, that you are failing at things, that is an adverse childhood experience that you will carry through your life. You will grow up with poor self-esteem and anxiety. (GP)

Suicidal ideation was also explicitly referenced.

Just general self-neglect as a consequence of depression associated with ADHD can have harmful effects. Even depression can lead to suicidal ideation and potential suicide risk or pseudo suicide risk and all of which can cause harm. (GP)

She (Granddaughter with ADHD) went through a little phase years ago threatening “I’ll kill myself,” and all that; I know a lot of kids do do that, but it seems to be the ones who are basically…Whether it is…ADHD or what, I don’t know. (Supporter of person with ADHD)

### Conceptualisation of ADHD in health

The second subtheme focusses on how participants conceptualised health within ADHD. A spectrum of conceptualisations emerged, ranging from what might be termed the dissolution of ADHD (ADHD is like any mental health issue or patients with ADHD are treated the same as *all* patients) to the siloing of ADHD (ADHD is distinct and not considered among other health issues).

No. (Laughs) I remember when I rang, they (primary care professional) were specifically like – I can’t remember the exact words, because it was a while ago, but it was something like, they were either saying, “Oh, we should look into that,” or, “Man, we really should have those available,” or something. They just didn’t have anything specific, I guess, for ADHD. They had the more generic mental health, but nothing ADHD-specific. (Person with ADHD)

But for me, it’s about health and social care and education, everyone working together to support children and young people, whatever the diagnosis, whatever the need. (GP)

In siloing of ADHD, some participants simply stated that they did not have the opportunity to discuss their wider health in the context of ADHD in primary care. In a related quote, a GP PM suggested that clinicians should be more vigilant for clusters of seemingly disparate physical health complaints.

No. My doctor is very; you go in for one specific thing, and then they just deal with that, rather than acknowledging how much health all is interlinked. (Person with ADHD)

for example, if somebody said that “look, I’ve got poor sleeping habits, I’m quite impulsive,” and they’ve got obesity, then perhaps you could say that somebody with ADHD, are these the signs of ADHD? (PM)

### Barriers to health in ADHD

This theme discusses factors that participants identified that affect health in ADHD.

#### Behavioural risks

Great emphasis was placed on what were termed ‘behavioural’ risks; the word ‘behaviour’ or phrase ‘risky behaviour’ was frequently used. Here, participants with lived and professional experience discussed generic ‘risky behaviour’ and further detailed the challenges of addiction, accidents and impulsive behaviour.

Yeah. Yeah, I am. I know that unmedicated it can shorten your life expectancy by 12 years because of the increased tendency to get into accidents, live a less healthy lifestyle and all that. (Person with ADHD)

I think that for people with ADHD… (pauses) (laughter) they tend to be a bit impulsive at times, so it is more of a challenge sometimes for them to take on board broader health messages. (GP)

but prior to being diagnosed a lot of people are using illicit drugs. That is a real concern. But all of these fit into the riskier behaviours. (PM)

Engagement with healthcare was only referenced as a factor in determining health by healthcare professionals exclusively. Healthcare professionals spoke of the challenges in engaging people with ADHD in health management and how sometimes simple things such as routine blood tests or attending appointments can be challenging for their patients with ADHD.

it’s just trying different ways of often pinning them (people with ADHD) down when we finally get them in the building to literally… There’s flags all over this: “Don’t let them leave without having a blood test, don't let them leave without…” (PM)

I don't know, a (did not attend) rate sometimes is really high for patients with ADHD, and it doesn’t matter how many times you invite them, they’ll still (did not attend) the next time. (PM)

#### Inadequate support

Some participants perceived support for health in ADHD to be inadequate with respect to the range, availability, accessibility and quality of services.

I think them being aware of—because I feel like ADHD treatment is almost like a tree. It’s like you start off with the diagnosis and normally medication. But then there’s so many different things you could do; cognitive behavioural therapy (CBT) is a really strong one, just general therapy I think is a really good one, and then support groups, talking to other ADHD people is super helpful. But when you go to a GP, they seem to only know the medication route, or the this, or the that. It’s quite limited. (Person with ADHD)

it is more of a challenge for them to keep to structures and appointments by systems that may not necessarily have much sympathy with their (people with ADHD) particular difficulties. (GP)

I suppose firstly recognise that there may be some additional needs, recognise that they (people with ADHD) may have (pause) as a consequence of an ADHD presentation some literacy, some educational, some access barriers to the practice. (GP)

Some participants felt that there was a lack of recognition of the wider health challenges and inequalities in relation to ADHD.

So essentially, you get this fantastic inequality of, if I’ve got a mental health disorder, I’m likely to die much younger than if I don’t have a mental health disorder. And that’s partly the impact of the mental health disorder, but much more, it’s the responsibility of the social decisions that you make, because you’re not getting the appropriate support and education that you need. (GP)

### Addressing health in ADHD

This theme was generated from responses where participants had suggested potential actions or responsibilities regarding the holistic management of ADHD.

A variety of primary care professionals were mentioned, including GPs, mental health workers (MHWs), health navigators, family support, social prescribers and university mental health services. Social prescribers were by far the most referenced form of wider healthcare support for people with ADHD, and more specifically in the context of personal independence payments and benefits and housing advice. However, social prescribing was not mentioned by lived experience participants.

the social prescriber is there and can help with things like occupational therapy, benefits, signpost them to various organisations, even give them that information with regards to what benefits they’re entitled to, all the day-to-day stuff. (PM)

I think it’s predominately around social prescribing support. So if they (person with ADHD) ask, or we feel that they have a specific challenge around the circumstances in which they live, which are exacerbating their challenges, then we do have a social prescriber that’s able to help with that. (GP)

MHWs were referenced by both participant groups. The form of support described by participants was that of generic mental health help with a problem-solving/brief intervention focused on a patient’s specific challenges.

I believe in the past I spoke to a specific support person at the practice. I was struggling to find somewhere to live, and they (mental health worker) were kind of associated with Mind, I think. (Person with ADHD)

It really is very person-centred, on what people want to bring. So it could be just holding space for someone, it could be whatever. So we do some coaching on identifying what their needs are. I think all the well-being workers work just in their individual way, but essentially yes, it’s coaching people to what they need, identifying support needs, goal-setting, signposting, a bit of coaching, emotional support. (MH)

Several participants with lived experience also reflected on the ways they thought peer support for ADHD could be useful.

I think if someone’s newly diagnosed, it can be quite overwhelming. So I think the support group aspect would be very important, because it’s a lot of people going, “You aren’t weird, that’s just what this is like”. Or, “You’re at this stage, and it will get better”. Or, “Here’s how I managed when I was at that stage”. That can be really helpful. (Person with ADHD)

#### Empowerment and enablement in ADHD

The concept of empowerment and enablement (or what might be termed self-efficacy in health psychology literature) was alluded to by several participants.^22^ Healthcare professionals discussed enabling or empowering patients to do things for themselves.

it’s perfectly possible for a social prescriber to recommend that to an ADHD patient and expect them to try and make the contact. Or if they thought that was something that that person would struggle to do, we can help them do that. (GP)

And we do want to empower our patients rather than help them, so that’s the ethos that we try and follow, so enable them to do things, we can do that. (GP)

Professionals also acknowledged that enabling people to do things for themselves is, in some cases, all that they are able to do within existing systems, and sometimes this can be a barrier to health.

And there is only so much we can do; we can’t go and pick them up and take them (to a specific physical health clinic). The link workers might get involved in that but the reality is we can’t do everything for people, so I think that is a potential barrier. (GP)

One person with lived experience also reflected on the challenge of empowerment as an approach, explaining the difficulties of taking on the task of navigating a service, particularly at a time when you are unwell.

it was a lot of, “Ah, well if you need this, then you need to go here and do this, do this. If you need this, you need to go here, do this, do this.” And then I didn’t wind up doing any of it because I was very ill and was struggling with task management. (Person with ADHD)

#### Specific recommendations

Some participants gave explicit recommendations for addressing wider health needs in ADHD. Lived experience participants talked about how didactic and pragmatic support could help with managing activities of daily living in ADHD.

To have more specific support relating how those symptoms might manifest in different situations and how you might want to manage it would probably be quite useful. Because I think it is quite difficult to map the broad symptoms into specific situations to be able to understand them in the minutiae. (Person with ADHD)

They also discussed better education for healthcare professionals around neurodiversity.

GPs reflected that flexibility in healthcare appointments and approaches can be beneficial when working with ADHD.

I would probably be more likely to write and say “This person has ADHD, they just couldn’t manage that; can we re-refer this person?” because of the two strikes and you’re out from a clinic. (GP)

one of my patients who’s an adult who’s known for a long time that she has ADHD, we work around her a little bit in that I know she’s a bit chaotic. So if she doesn’t turn up, I’m not like “Oh my god,” (laughter) I might ring her instead and say “I was expecting to see you today but can we deal with it on the phone?” (GP)

## Discussion

This study aimed to explore attitudes and understandings of the wider health needs of adults with ADHD from the perspectives of primary care professionals and those with lived experience.

Three themes were generated: understanding health in ADHD, barriers to health in ADHD and addressing health in ADHD.

To dissect our first theme, understanding health in ADHD*,* the identified health risks are consistent with those seen elsewhere in the literature; it is well-documented that there are greater risks of physical and mental health problems, low self-esteem and suicide.^514 23^,^26^ However, the findings demonstrate some awareness of these health risks among healthcare professionals and those with lived experience, representing an extension of previous primary care ADHD research, which has reported only a general lack of confidence and understanding in holistic ADHD care.^27 28^

Within this theme, we then capture how ADHD is conceptualised in relation to health problems, where the data suggest two processes along a spectrum in which either ADHD does not present specific challenges in healthcare access and utilisation (ie, our practice provides X, ‘as for all patients’ or ‘for all mental health patients’) or that ADHD care becomes ultra-specified, such that ADHD is considered in isolation, and there are limited opportunities to discuss the multiple health implications of ADHD (ie, participants who discussed the ‘one issue per appointment’ problem).

First, it is important to note that this data highlights some of the tensions caused by practicing under significant time and resource constraints, and to conclude that GPs need to change/adapt their approach would be overly simplistic. However, given the context of the study and data, they provide some useful insights into understanding how to better integrate mental and physical healthcare within ADHD.

The concept of mental and physical health integration in primary care is not novel. For example, there is a well-defined framework for considering physical health in the context of severe mental illness (considering the cardiometabolic effects of antipsychotics), which now has associated quality and outcomes framework (QOF) points available (QOF points being incentivised auditable standards for GP practices to adhere to, based on National Institute for Health and Care Excellence guidance).^29^ Such a framework is currently lacking for ADHD, aside from basic checks for those prescribed medications (eg, monitoring height and weight; risks if using additional substances). Even in medication checks, there is significant variation with respect to shared care agreements between primary and secondary care and who is responsible for medication-related checks.^3 30^

Currently, there is insufficient data to support further QOF points for ADHD management surrounding health management, given the lack of evidence-based monitoring and interventions. In autism research, where healthcare outcomes and healthcare experience have been better synthesised, intervention development can occur, as demonstrated by the co-design of an annual autism health check.^31^

Behavioural risks were seen as heterogeneous; they included references to addiction, risky behaviour and accidents, all of which are consistent and well-researched in the ADHD literature.^32^,^36^ Participants identified the importance of timely diagnosis and appropriate treatment in mitigating these behavioural risks (eg, ‘prior to being diagnosed, a lot of people are using illicit drugs’). This view is supported by large epidemiological studies from Swedish Registry Data examining the impact of stimulant medication in the reduction of various outcomes, including substance use and criminal recidivism.^37 38^ However, there is currently little data to inform psychosocial interventions (such as those that may be afforded in a non-specialist primary care setting) within health risk reduction (Ward *et al*, under review). Consequently, there exists a population of people who are unable to access either pharmacological or psychosocial interventions.

Healthcare professionals also expressed frustrations about engaging people with ADHD in health, mainly with respect to attending appointments. While there is unclear quantitative evidence of appointment attendance in adults with ADHD, one study has shown that children with ADHD (and their parents) were more likely to miss outpatient appointments than other psychiatric patients.^39^ Missed appointments and mitigations are covered in more depth in related MAP study work in preparation.^18^

A sentiment captured well by ‘capacity and capability are not the same’, from one healthcare professional, shows a thread that ran through this theme. Both people with ADHD and professionals perceived that while those with ADHD would be ‘capable’ of booking appointments or otherwise engaging with healthcare, complex processes in dysfunctional systems complicated this task.^3 40 41^

This ties into the empowerment and enablement subtheme within addressing health, where healthcare professionals discuss wanting to ‘empower’ and ‘enable’ people with ADHD in their health behaviour, while conversely, it is recognised that the system is not ‘sympathetic’ to the challenges healthcare structures may pose to people with ADHD and that sometimes more granular didactic support is required.

Since the 1970s, self-efficacy has been within the vocabulary of health psychology as a framework through which people may evaluate their ability to perform a function.^22 42^ Within ADHD, Newark and colleagues found lower self-efficacy, related to poorer self-esteem.^43^ Furthermore, Enggaard and colleagues explored guided self-determination with adolescents with chronic conditions to devise strategies to improve health behaviour.^44^ While decades of health psychology show that self-efficacy is important in the adoption of health behaviours, the core principles of self-efficacy include both personal and vicarious experiences of success. In a healthcare system that, according to this study, does not routinely or systematically support people with ADHD with their wider health, self-efficacy is much harder to establish as a concept.

In this vein, inadequate support was also frequently referenced with respect to health challenges, which in turn led to the identified healthcare professional’s health within our addressing health theme. Those with lived experience referred to the notion that there was limited holistic support for ADHD, only medication, and furthermore, support did not consider the breadth of health challenges faced. This is somewhat contrary to what is reflected by healthcare professionals, who frequently referred particularly to social prescribers and mental health support workers, who were described as pluripotent in what they could offer, particularly with respect to social assistance (housing and benefits) and mental health support (counselling, goal setting and behavioural therapy). In fact, great emphasis was placed on the importance of social support by healthcare professionals, who recognised that people with ADHD may struggle with literacy and educational attainment and frequently referenced how useful social prescribers are in helping patients access housing support and government benefits.

The data do not provide any further insights into this discrepancy. The limited qualitative research exploring the factors surrounding implementing social prescribing suggests that its success is contingent on proper role definition, integration within primary care and development with patient input.^45^,^47^ While the same literature does describe the benefit of the flexibility afforded by social prescribing, it also shows that the flexibility needs to be crafted around a clear framework to be accessible, or else it becomes frustrating for the staff offering it and the patients accessing it. This links with a recommendation made by one participant for situation-specific support, where the skills of social prescribing would be well-suited to adapting to situations in everyday life. There appears to be a gap in the evidence regarding the tailoring of social prescribing for people with ADHD (eg, help accessing disability-related financial support).

### Strengths and limitations

The strength of the study is the diverse range of experiences it has captured, recruiting primary care professionals and patients/supporters from varied backgrounds. Furthermore, our study has taken a broad conceptualisation of health beyond traditional pharmacological and non-pharmacological support within ADHD, including a holistic appreciation of the health challenges routinely faced by people with ADHD in a setting where they may expect to receive help. This study also helps to highlight that health inequalities are context-dependent, and addressing structural, service and knowledge gaps is key to addressing the issue.^3 17^ This study is also useful in identifying challenges that could form the basis of future coproduction work by the study authors.^48^

While our work is only a preliminary insight into the qualitative experiences of holistic health in ADHD, more in-depth discussion regarding the specific health challenges within ADHD and a better dissection of the intersectional identities of participants may advance future work in this field. One limitation of our study is that it treats ADHD management in England as a singular entity. Our recruitment was framed within the ICS structure adopted in NHS England commissioning and policy and recognises the significant heterogeneity in practice that stems from this but does not include data from other countries in the UK. This study provides an important oversight of generalised function and dysfunction for this condition but is strongly contextualised within the ICS system design, specific to NHS England, with approaches tailored to local resources and needs.

## Conclusions

This study set out to explore perspectives on the broader health needs of people with ADHD from the point of view of primary care professionals and people with lived experience. Findings suggest that there is recognition of a spectrum of needs, including risky behaviours, co-occurring conditions, self-care and self-neglect and challenges in engagement with health services. Participants also recognised the role of wider socioeconomic factors affecting the health of people with ADHD. Our findings suggest that there is both support for, and a gap in, a more structured approach to holistic management of the health of people with ADHD. Further research should focus on the exploration, definition, evaluation and implementation of effective prevention and intervention strategies.

## supplementary material

10.1136/bmjopen-2023-083539 online supplemental file 1

## Data Availability

Data are available upon reasonable request.

## References

[1] Cortese S, Sabé M, Chen C (2022). Half a century of research on attention-deficit/hyperactivity disorder: a scientometric study. Neurosci Biobehav Rev.

[2] National Institue for Health and Care Excellence (2019). Recommendations | Attention deficit hyperactivity disorder: diagnosis and management | Guidance | NICE. https://www.nice.org.uk/guidance/ng87/chapter/Recommendations.

[3] Young S, Asherson P, Lloyd T (2021). Failure of healthcare provision for attention-deficit/hyperactivity disorder in the United Kingdom: a consensus statement. Front Psychiatry.

[4] Boilson M, Shah P, MacIver I (2017). ADHD in adults: good practice guidelines.

[5] Pan PY, Bölte S (2020). The association between ADHD and physical health: a co-twin control study. Sci Rep.

[6] Faraone SV, Banaschewski T, Coghill D (2021). The world federation of ADHD international consensus statement: 208 evidence-based conclusions about the disorder. Neurosci Biobehav Rev.

[7] Du Rietz E, Brikell I, Butwicka A (2021). Mapping phenotypic and aetiological associations between ADHD and physical conditions in adulthood in Sweden: a genetically informed register study. Lancet Psychiatry.

[8] Semeijn EJ, Sandra Kooij JJ, Comijs HC (2013). Attention-deficit/hyperactivity disorder, physical health, and lifestyle in older adults. J Am Geriatr Soc.

[9] Chen M-H, Pan T-L, Hsu J-W (2018). Risk of type 2 diabetes in adolescents and young adults with attention-deficit/hyperactivity disorder: a nationwide longitudinal study. J Clin Psychiatry.

[10] Chen VCH, Chan HL, Wu SI (2022). Methylphenidate and mortality in children with attention-deficit hyperactivity disorder: population-based cohort study. Br J Psychiatry.

[11] Nigg JT (2013). Attention-deficit/hyperactivity disorder and adverse health outcomes. Clin Psychol Rev.

[12] Stickley A, Koyanagi A, Takahashi H (2017). Attention-deficit/hyperactivity disorder and physical multimorbidity: A population-based study. Eur Psychiatry.

[13] Barker ED, Ing A, Biondo F (2021). Do ADHD-impulsivity and BMI have shared polygenic and neural correlates?. Mol Psychiatry.

[14] Brook JS, Brook DW, Zhang C (2013). Adolescent ADHD and adult physical and mental health, work performance, and financial stress. Pediatrics.

[15] London AS, Landes SD (2016). Attention deficit hyperactivity disorder and adult mortality. Prev Med.

[16] Landes SD, London AS (2018). Self-reported ADHD and adult health in the united states. https://doi.org/101177/1087054718757648.

[17] Asherson P, Leaver L, Adamou M (2022). Mainstreaming adult ADHD into primary care in the UK: guidance, practice, and best practice recommendations. BMC Psychiatry.

[18] Price A, Smith JR, Mughal F (2023). Protocol for the mixed methods, Managing young people (aged 16–25) with Attention deficit hyperactivity disorder in Primary care (MAP) study: mapping current practice and co-producing guidance to improve healthcare in an underserved population. BMJ Open.

[19] Byrne D (2022). A worked example of Braun and Clarke’s approach to reflexive thematic analysis. Qual Quant.

[20] Braun V, Clarke V (2019). Reflecting on reflexive thematic analysis. Qual Res Sport Exerc Health.

[21] Malterud K, Siersma VD, Guassora AD (2016). Sample size in qualitative interview studies: guided by information power. Qual Health Res.

[22] Bandura A (1977). Self-efficacy: toward a unifying theory of behavioral change. Psychol Rev.

[23] Harpin V, Mazzone L, Raynaud JP (2016). Long-term outcomes of ADHD: a systematic review of self-esteem and social function. J Atten Disord.

[24] Crowell SE, Derbidge CM, Beauchaine TP, Nock MK (2014). The Oxford handbook of suicide and self-injury.

[25] Hinshaw SP, Owens EB, Zalecki C (2012). Prospective follow-up of girls with attention-deficit/hyperactivity disorder into early adulthood: continuing impairment includes elevated risk for suicide attempts and self-injury. J Consult Clin Psychol.

[26] Stickley A, Koyanagi A, Ruchkin V (2016). Attention-deficit/hyperactivity disorder symptoms and suicide ideation and attempts: Findings from the Adult Psychiatric Morbidity Survey 2007. J Affect Disord.

[27] French B, Perez Vallejos E, Sayal K (2020). Awareness of ADHD in primary care: stakeholder perspectives. BMC Fam Pract.

[28] French B, Sayal K, Daley D (2019). Barriers and facilitators to understanding of ADHD in primary care: a mixed-method systematic review. Eur Child Adolesc Psychiatry.

[29] NHS England (2023). Quality and outcomes framework guidance for 2023/24. https://www.england.nhs.uk/publication/quality-and-outcomes-framework-guidance-for-2023-24/.

[30] Price A, Ford T, Janssens A (2020). Regional analysis of UK primary care prescribing and adult service referrals for young people with attention-deficit hyperactivity disorder. BJPsych Open.

[31] Taylor H, Ingham B, Mason D (2023). Co-design of an NHS primary care health check for autistic adults. Autism.

[32] Shoham R, Sonuga-Barke E, Yaniv I (2021). What drives risky behavior in ADHD: insensitivity to its risk or fascination with its potential benefits?. J Atten Disord.

[33] Dekkers TJ, de Water E, Scheres A (2022). Impulsive and risky decision-making in adolescents with attention-deficit/hyperactivity disorder (ADHD): The need for a developmental perspective. Curr Opin Psychol.

[34] Graziano PA, Reid A, Slavec J (2015). ADHD symptomatology and risky health, driving, and financial behaviors in College. J Atten Disord.

[35] Evren B, Evren C, Dalbudak E (2018). Relationship of internet addiction severity with probable ADHD and difficulties in emotion regulation among young adults. Psychiatry Res.

[36] Treur JL, Demontis D, Smith GD (2021). Investigating causality between liability to ADHD and substance use, and liability to substance use and ADHD risk, using Mendelian randomization. Addict Biol.

[37] Chang Z, Lichtenstein P, Halldner L (2014). Stimulant ADHD medication and risk for substance abuse. J Child Psychol Psychiatry.

[38] Lichtenstein P, Halldner L, Zetterqvist J (2012). Medication for attention deficit-hyperactivity disorder and criminality. N Engl J Med.

[39] Nomura K, Tarumi R, Yoshida K (2021). Cancellation of outpatient appointments in patients with attention-deficit/hyperactivity disorder. PLoS ONE.

[40] Kozlowska O, Lumb A, Tan GD (2018). Barriers and facilitators to integrating primary and specialist healthcare in the United Kingdom: a narrative literature review. *Future Healthc J*.

[41] Baker C (2023). NHS key statistics: england, july 2023. https://commonslibrary.parliament.uk/research-briefings/cbp-7281/.

[42] Lawrance L, McLeroy KR (1986). Self-efficacy and health education. J Sch Health.

[43] Newark PE, Elsässer M, Stieglitz RD (2012). Self-esteem, self-efficacy, and resources in adults with ADHD.

[44] Enggaard H, Laugesen B, DeJonckheere M (2021). Impact of the guided self-determination intervention among adolescents with co-existing ADHD and medical disorder: a mixed methods study. Issues Ment Health Nurs.

[45] Chng NR, Hawkins K, Fitzpatrick B (2021). Implementing social prescribing in primary care in areas of high socioeconomic deprivation: process evaluation of the “Deep End” community Links Worker Programme. Br J Gen Pract.

[46] Rhodes J, Bell S (2021). “‘It sounded a lot simpler on the job description’”: a qualitative study exploring the role of social prescribing link workers and their training and support needs (2020). Health Soc Care Community.

[47] Simpson S, Furlong M, Giebel C (2021). Exploring the enablers and barriers to social prescribing for people living with long-term neurological conditions: a focus group investigation. BMC Health Serv Res.

[48] MAP Study (2024). Co-production – MAP Study.

[49] Gudka R, Becker K, Ward J (2024). Primary care provision for young people with ADHD: a multi-perspective qualitative study. Br J Gen Pract.

