# Supplementary material for: Wider health needs in attention deficit hyperactivity disorder from lived and professional experience: a qualitative framework analysis
Source: BMJ Open. 2024 Aug 14;14(8):e083539. doi: 10.1136/bmjopen-2023-083539 (PMC11331868; doi:10.1136/bmjopen-2023-083539)
Supplement: Supplementary file 2 [file bmjopen-2023-083539.reviewer_comments.pdf]

PEER REVIEW HISTORY

BMJ Open publishes all reviews undertaken for accepted manuscripts. Reviewers are asked to complete a checklist review form and are provided with free text boxes to elaborate on their assessment. These free text comments are reproduced below.

ARTICLE DETAILS

**TITLE (PROVISIONAL)**	Wider health needs in attention deficit hyperactivity disorder from lived and professional experience: a qualitative framework analysis
**AUTHORS**	Ward, John; McBride, Audrey; Gudka, Rebecca; Becker, Kieran; Newlove-Delgado, Tamsin; Price, Anna

VERSION 1 - REVIEW

**REVIEWER NAME**	Maeir, Adina
**REVIEWER AFFILIATION**	Hebrew University of Jerusalem
**REVIEWER CONFLICT OF INTEREST**	I have no competing interests
**DATE REVIEW RETURNED**	14-Jan-2024

**GENERAL COMMENTS**	Thank you for the opportunity to review this important study.<br>the authors address a serious gap in research and practice - i.e. providing integrative holistic care for individuals with ADHD.<br>The qualitative methodolgy was effective in exposing the problem, the barriers and potential solutions. I am looking forward to the next phase: developing an integrative care plan, using a codesign method with all relevant sakeholders.<br>congratulations on your significant contribution to understanding the real world needs of this large populations.<br>my only reservation is that a COREQ (COnsolidated criteria for REporting Qualitative research) Checklist was not included.<br>I leave this for the editor to decide if it is required.

**REVIEWER NAME**	Poulton, Alison
**REVIEWER AFFILIATION**	University of Sydney, Sydney Medical School Nepean
**REVIEWER CONFLICT OF INTEREST**	Personal fees and non-financial support from Shire/Takeda, and book royalties from Disruptive Publishing (ADHD Made Simple).
**DATE REVIEW RETURNED**	27-Feb-2024

**GENERAL COMMENTS**	It is important when research is carried out on particular groups of people that the views of those with lived experience are sought. This is true for the topic of research, the design of the study and the way that the research is interpreted and presented. The researchers are investigating the perspectives of people with ADHD and their supporters and providers of primary health care.<br><br>There is a lot that is missing about the design of the study. For example the use of study research advisory groups (RAGs): How were they recruited? How much did their views influence the five key areas? How many people per group? How often did they meet and communicate with the research team? Was there any overlap between them and the research participants? Were the study questions modified after consultation with the RAGs? Were the

	study questions piloted first? Which members of the research team carried out the interviews? What was the professional background of the interviewers? Did they undertake any interview training? Did the participants have the opportunity to review the transcripts and provide feedback on findings? Did any members of the research team have lived experience of ADHD?<br><br>In this small study 'Supporters were recruited via the people with ADHD who participated in the study. 20 participants were recruited, consisting of five general practitioners (GPs), five practice managers (PMs), one mental health worker (MHW), six people with ADHD (YP) and three supporters of people with ADHD' This suggests that of the 14 supporters all would relate to only 6 individuals with ADHD. Is this correct?<br>There is no mention of data saturation using this small sample. How was this assessed?<br>Is the framework matrix available for review?<br><br>The study was ethically approved but there is no detail about the process of consent.<br><br>The researchers state 'The strength of the study is the diverse range of experiences it has captured, recruiting primary care professionals and patients/supporters from varied backgrounds' but give almost zero demographic data. Therefore their diversity cannot be evaluated by the reader.<br><br>The data are perhaps overinterpreted, with other explanations not discussed. For example, regarding the conceptualisation of ADHD the authors have described the following extremes: 'the data suggest two processes along a spectrum in which either ADHD does not present specific challenges in healthcare access and utilisation (i.e. our practice provides X, 'as for all patients' or 'for all mental health patients'), or that ADHD-care becomes ultraspecified, such that ADHD is considered in isolation and there are limited opportunities to discuss the multiple health implications of ADHD (i.e. participants who discussed the 'one issue per appointment' problem).'<br><br>Rather than this being a philosophical or conceptual matter, might it be more related to the role of the worker: that the GP is more conscious of the time allocated to each appointment and the patients waiting to be seen – while the practice manager can perhaps afford to criticize the time-poor GP? This explanation fits with the examples given:<br><br>'A GP practice manager suggested that perhaps clinicians should be more vigilant for clusters of seemingly disparate physical health complaints.' By contrast the perception about the GP: "No. My doctor is very; you go in for one specific thing, and then they just deal with that, rather than acknowledging how much health all is interlinked."<br><br>So this difference in outlook may have a mundane, practical cause rather than a particular conceptualization leading to ADHD being either ignored and focus being on the other medical problems or the doctor's personal preference to treat ADHD in isolation.

	The message overall is important but already well established in (probably) every setting where it has been studied – that people with ADHD have difficulty getting their health needs addressed.<br><br>Some solutions would be good – for example having more consistency in the availability and skillsets of 'social prescribers' who could provide support with developing life skills and navigating the health systems.<br><br>Ref 9 – published 2019 – listed incorrectly 'in press'

**REVIEWER NAME**	Janlov, Ann-Christin
**REVIEWER AFFILIATION**	Kristianstad University College, Faculty of Health Science Kristianstad university
**REVIEWER CONFLICT OF INTEREST**	I have no competing interests.
**DATE REVIEW RETURNED**	29-Feb-2024

**GENERAL COMMENTS**	This is a very important and well needed study with potential. However, the main weakness is the stringency and transparency of the study, and that the qualitative methodological research process needs significant improvement. All methodological aspects must be described for possibilities to replicate the study. Please see COREQ (COnsolidated criteria for REporting Qualitative research) Checklist.<br>Abbreviations: Some need better clarification<br>People with (lived experience of) ADHD. Suggest you exclude lived experience. YP meaning what? Which are the ADHD supporters? What is YP?<br>Problem and background: I consider well written and easy to grasp.<br>Background: Page 4, 47: … a greater exposure to a range of health risks… in comparison to?<br>4, 50-54… in comparison to) Reference 5-8 is not sufficient enough.<br>Scoping review (14) There is no reference in the text, and in the References is given BMJ Open 13:e068184, meaning?<br><br>Aim: Adequate. However, the Purpose/aim is differently formulated in the manuscript. It is important to use the same formulation to avoid that something new is excluded or included. Moreover, I advise you to use just "experience" or "personal experience" instead of "lived experience" since the latter is associated to specific qualitative approaches/methods.<br>Method<br>Following aspects (see below) should be clarified in detail to strengthen the trustworthiness (see Lincoln & Guba (1985) of the study and possibility to replicate the study.<br>Design: A qualitative approach/method…. Clarify your theoretical assumptions and positioning. Inductive/ deductive approach? Manifest/latent level of interpretation?<br>References are needed.<br>Participants: Type of sample, recruiting procedure in detail, numbers of each participant group separately. Primary care professionals (number of GPs, number of Practice managers, number of Allied health care professionals (such as?), number of Persons/indiviuals/patients with individual/personal experience (or preferably, just use experience of) and number of supporters/carers. A clarifying table over the demographics of the participants is needed.<br>Data collection: How the data was collected is unclear.

	More details about the interviews are needed. Specify the type of interviews, number of interviews, the number of participants, and the composition of participants per interview.<br>Please provide a table with the interview guides and the research questions. Time of the data collection?<br>Analysis:<br>The given reference of the used method of analysis is confusing. The text describes the use of Byrne (2022) [15, 16] and (Braun and Clark and Gale et al, 2013). In the list of references Braun and Clarke (2006) is given. It is not sufficient to provide an article with how Byrne handled the RTA. What of the analysis processes have you used and how? What is the difference between the TA of Braun and Clarke (2006) and later Braun and Clarke RTA. Please clarify. Please provide a table over the analysis process to improve the transparency of the analysis. All the way from meaning unit (do not forget to include what interview, participant/number) to subthemes and themes.<br>The writing “Reflective journals were kept aiding the process of coding and theme generation”, in what way?<br>Time of the analysis in relation to the data collection?<br><br>Results<br><br>I find the results difficult to grasp. The themes and subthemes seem adequate.<br>I recommend a short introduction /ingress for the reader which also describe how the quotes should be understood. What interview? Is it a single participant or more? Patient or GP in what interview?<br><br>The result consists of too many quotes. This way the result becomes too fragmented. What does YP and S mean? You need to clarify more in the quotes. In brackets you can insert a clarification of as for example “they” (patients) or “we” (GPs) for a better understanding of the quote. As a whole convert more of them to describing text.<br><br>Table 1. The presentation of Themes and subthemes<br>A subtheme in the table is missing in the result section, Peer Dynamics.<br>Theme description. "Their" refers to whom?<br><br>Discussion<br>The discussion needs revision.<br>The discussion fails to appear when lifting aspects to discuss related to your own result and thereafter just write/refer to numbers in the list of references as (28,28, 39).<br><br>List of references<br>Please provide DOI when possible<br><br>Contributions:<br>A reminder. In a qualitative study with inductive approach, you do not generate hypothesis.

VERSION 1 – AUTHOR RESPONSE

Reviewer 1 Comments

Thank you for the opportunity to review this important study.<br>the authors address a serious gap in research and practice - i.e. providing integrative holistic care for individuals with ADHD.<br>The qualitative methodolgy was effective in exposing the problem, the barriers and potential solutions. I am looking forward to the next phase: developing an integrative care plan, using a codesign method with all relevant sakeholders.<br>congratulations on your significant contribution to understanding the real world needs of this large populations.<br>my only reservation is that a COREQ (COnsolidated criteria for REporting Qualitative research) Checklist was not included.<br>I leave this for the editor to decide if it is required	Thank you for your praise of our work. We have included a COREQ checklist as you have suggested.	
**Reviewer 2**		
There is a lot that is missing about the design of the study. For example the use of study research advisory groups (RAGs):<br><br>How were they recruited?<br><br>How much did their views influence the five key areas?<br><br>How many people per group?<br><br>How often did they meet and communicate with the research team?	Thank you for this comment. We have now included more detail on our RAGs, and methodology which we hope will provide more insight.<br><br>We have now included a reflexive statement.	Methods Section (pages 7-9)<br><br>In total, 20 participants were recruited, consisting of five general practitioners (GPs), five practice managers (PMs), one mental health worker (MHW), six people with ADHD (P) and three supporters of people with ADHD (S). Demographics of the participants are given in Table 1.

Was there any overlap between them and the research participants?<br><br>Were the study questions modified after consultation with the RAGs?<br><br>Were the study questions piloted first?<br><br>Which members of the research team carried out the interviews?<br><br>What was the professional background of the interviewers?<br><br>Did they undertake any interview training?<br><br>Did the participants have the opportunity to review the transcripts and provide feedback on findings?<br><br>Did any members of the research team have lived experience of ADHD?		Interview Methodology<br><br>Interviews were conducted virtually (by JW, RG, KB, TND and AP), given the geographic spread of this study. Interviews took place between March and June 2023. They were either conducted securely through Microsoft Teams (MS Teams) or by mobile phone depending on participant preference. Participants' electronic written consent was taken via email prior to interviews and reconfirmed at the time of interview. Participants were compensated for their time with a £25 voucher.<br><br>RG, TND and AP already had qualitative interview experience. JW and KB therefore received training on the topic guide and qualitative interviewing from AP and RG. The professional backgrounds of the interviewers were; clinical academics (JW, TND), student researchers (RG, KB) and research fellow from psychology background (AP).<br><br>Interviews were semi-structured and approximately one hour in length, and each participant only participated in one interview. Apart from one interview, with two professional interviewees, all participants partook in one-to-one interviews with a researcher.<br><br>Interviews were recorded, transcribed verbatim by a trusted service and deleted. Participants did not see transcripts post-interview.

		Interviews were up to one hour long and based on pre-determined topic guides. These topic guides reflected the study aims and were developed in conjunction with the study research advisory groups (RAGs), ensuring patient and public involvement and engagement (PPIE). The topic guides were also piloted within RAGs. Two versions were created, with one tailored towards primary care professionals and the other tailored for those with lived experiences (i.e., people with ADHD and their supporters). The topic guides covered five key areas; how people with ADHD access primary care, healthcare support in primary care (including pharmacological and non-pharmacological support), healthcare and support for wider healthcare needs through primary care, information and resources and digital solutions to help manage ADHD, and final reflections. The questions relating to this paper are given in Table 2, whilst the full topic guide is available in supplementary materials.<br><br>Research Advisory Group (RAG)<br><br>There were two research advisory groups for this study; a healthcare professional group (n=7) and a lived experience group (n=8). Our healthcare professional group consisted of an ADHD specialist nurse, an occupational therapist, three GPs. Our lived experience group comprised

		of 5 young people aged between 17-25, from diverse ethnic backgrounds across England, and three parents of young people with ADHD. None of the RAG participants took part in the study.<br><br>Four meetings for each group were held at approximately 6 monthly intervals through the project. Following RAG involvement, it was noted that particularly our lived experience participants were very encouraging of our questions regarding health and ADHD.<br><br>Early qualitative findings from this project were shared with the RAGs, in line with the plans for the further development of this work.<br><br>Qualitative Analysis<br><br>For this project, we used reflexive thematic analysis, using framework methods in the manner described in Byrne (2022), using a constructionist rather than positivist framework [20–23]. This was undertaken between July and August 2023.<br><br>A preliminary framework was developed using an inductive approach to the data. This involved line-by-line coding of initial transcripts by researchers (AP, RG, JW, KB) and code refinement of all transcripts. Data collated under codes relating to perceptions of broader health and healthcare needs in ADHD were identified and reviewed, and a framework

		matrix was created. Data coded under other codes were reviewed to ensure no relevant data had been missed. Researchers then produced column summaries which were used to generate themes and subthemes relating to this research question.<br><br>Reflexive Statement<br><br>The professional backgrounds of the interviewers were; clinical academics (JW, TND), student researchers (RG, KB) and research fellow from psychology background (AP). In respect to previous and lived experience, AP is a parent of a young person with ADHD, which, combined with involvement in 92 interviews with young people with ADHD and their supporters (in her role as a researcher) has shaped her approach. She also works closely with and has been involved in 52 interviews with healthcare professionals supporting people with ADHD in secondary care settings. Throughout the study AP held regular meetings with the team to facilitate reflections on underlying assumptions/presuppositions about the sample and topic area. Reflective journals were kept to aid the process of coding and theme generation, by highlighting and separating the personal engagement of researchers with the study and its findings (e.g. clinical

		academics dealing with criticism of clinical services).<br><br>Reflective journals were kept to aid the process of coding and theme generation, by highlighting and separating the personal engagement of researchers with the study and its findings (e.g. clinical academics dealing with criticism of clinical services).<br><br>Public Involvement and Engagement (PPIE)<br><br>Patient public involvement and engagement (PPIE) was used in the development of the original MAP Study protocol [24], as well as in the development of the topic guide for this qualitative study.
In this small study 'Supporters were recruited via the people with ADHD who participated in the study. 20 participants were recruited, consisting of five general practitioners (GPs), five practice managers (PMs), one mental health worker (MHW), six people with ADHD (YP) and three supporters of people with ADHD' This suggests that of the 14 supporters all would relate to only 6 individuals with ADHD. Is this correct?	Thank you, we have now clarified our text to communicate more clearly that supporters (n=3) were defined as people directly supporting the person with ADHD, such as a partner, close friend or relative. There were 6 people with ADHD in the study, and 3 supporters. The remainder were primary care professionals.	Methods (page 7)<br><br>Supporters (e.g., parents/friends) were recruited via the people with ADHD who participated in the study.<br><br>In total, 20 participants were recruited, consisting of five general practitioners (GPs), five practice managers (PMs), one mental health worker (MHW), six people with ADHD (P) and three supporters of people with ADHD (S).
There is no mention of data saturation using this small sample. How was this assessed?	Our purposive sampling strategy was chosen to select information-rich cases. We chose to use the concept of information power, taking into account the specific nature of our	Methods (page 9)<br><br>A preliminary framework was developed using an inductive approach to the data, and assessing information power

	sample and focussed study aim. As we progressed with analysis, we continued to review the information power in terms of the richness and specificity of material and the contribution of new insights from our data, rather than aiming for "complete description of all aspects of a phenomenon" which is often associated with assertion of data saturation, and which is more closely associated with grounded theory rather than our critical realist approach. We have added text to this effect in the Methods.	as analysis progressed (Malterud et al., 2016).
Is the framework matrix available for review?	Thank you for this comment. We hope that given our clearer communication of our methods, this is no longer required. If this is still required, this is something that would need to be discussed with other collaborators within the wider study team, as we're sure you can appreciate given the sensitive nature of such data.	
The study was ethically approved but there is no detail about the process of consent.	Thank you. We have included a consent statement, and updated our methods.	Methods (Interview Methodology) (Page 7)<br><br>Participants' electronic written consent was taken via email prior to interviews and reconfirmed at the time of interview.
The researchers state 'The strength of the study is the diverse range of experiences it has captured, recruiting primary care professionals and patients/supporters from varied backgrounds' but give almost zero demographic data. Therefore their diversity cannot be evaluated by the reader.	Thank you for this comment. We have added further demographic information (including a Table) as well as included greater information on the diversity of the practices included in this study.	Methods (Page 6/7)<br><br>"Four of these practices were urban, and one was rural. Three of these practices were in areas of high deprivation, and one of these practices served a large student population."<br><br>"In total, 20 participants were recruited, consisting of five general practitioners (GPs),

		five practice managers (PMs), one mental health worker (MHW), six people with ADHD (P) and three supporters of people with ADHD (S). Demographics of the participants are given in Table 1.”
The data are perhaps overinterpreted, with other explanations not discussed. For example, regarding the conceptualisation of ADHD the authors have described the following extremes: ‘the data suggest two processes along a spectrum in which either ADHD does not present specific challenges in healthcare access and utilisation (i.e. our practice provides X, ‘as for all patients’ or ‘for all mental health patients’), or that ADHD-care becomes ultra-specified, such that ADHD is considered in isolation and there are limited opportunities to discuss the multiple health implications of ADHD (i.e. participants who discussed the ‘one issue per appointment’ problem).’<br><br>Rather than this being a philosophical or conceptual matter, might it be more related to the role of the worker: that the GP is more conscious of the time allocated to each appointment and the patients waiting to be seen – while the practice manager can perhaps afford to criticize the time-	Thank you for this comment, and we appreciate that you are engaging with the themes we have raised in our manuscript. In essence, you have highlighted something we hope is evident from the paper, that ADHD does not perhaps marry up well with existing healthcare structures (particularly for adults with ADHD).<br><br>Some of us are clinicians ourselves, and we are very aware of the time pressures on clinicians that exist when seeing patients in primary care. However, this study is not looking at how people are acting in consultations—we accept, as with any qualitative research, that what people reflect in a qualitative interview would likely be different from their clinical practice anyway. Therefore, in this frame—an interview about approaching patients—we feel that it is noteworthy that there were differences in how different perspectives on health and ADHD were relayed.<br><br>Furthermore, as part of our reflexivity, we have to accept that whilst we may disagree with what a participant has said about their GP being for ‘one thing’, it is not for us to change the meaning of that quote because of our understanding about the healthcare system.<br><br>In recognition of this point, we have included a sentence in our discussion	Discussion (pages 19/20)<br><br>Firstly, it is important to note that this data highlights some of the tensions caused by practicing under significant time and resource constraints, and to conclude that GPs need to change/adapt their approach would be overly simplistic. However, given the context of the study and data, they provide some useful insights into understanding how to better integrate mental and physical healthcare within ADHD.

poor GP? This explanation fits with the examples given:<br><br>‘A GP practice manager suggested that perhaps clinicians should be more vigilant for clusters of seemingly disparate physical health complaints.’ By contrast the perception about the GP: "No. My doctor is very; you go in for one specific thing, and then they just deal with that, rather than acknowledging how much health all is interlinked."<br><br>So this difference in outlook may have a mundane, practical cause rather than a particular conceptualization leading to ADHD being either ignored and focus being on the other medical problems or the doctor’s personal preference to treat ADHD in isolation.	to ensure that our point is not read as overly-critical of individual practice working under significant pressure.	
The message overall is important but already well established in (probably) every setting where it has been studied – that people with ADHD have difficulty getting their health needs addressed.	Thank you for this comment. We agree that there is lots of literature looking at healthcare access, but these primarily look at ADHD-related care. The intersection with physical and mental health needs within ADHD, and how these are viewed from primary care, has not previously (to our knowledge) been addressed in this way.	
Some solutions would be good – for example having more consistency in the availability and skillsets of ‘social prescribers’ who could provide support with developing life skills and	Thank you for this comment. This relates back to our discussion of framework development; we cannot intervene without a framework to design interventions on.	Discussion, Page 24<br><br>This study is also useful in identifying challenges that could form the basis of future coproduction work by the study authors [51]

navigating the health systems.	Related work as part of the MAP study has involved co-production research to review evidence from this study (amongst other evidence) and focusses on identifying simple solutions. This is in process. We have preliminary resources available on our website. https://sites.exeter.ac.uk/mapadhd/co-production/	
Ref 9 – published 2019 – listed incorrectly 'in press'	Thank you. We have corrected this.	
**Reviewer 3**		
This is a very important and well needed study with potential. However, the main weakness is the stringency and transparency of the study, and that the qualitative methodological research process needs significant improvement. All methodological aspects must be described for possibilities to replicate the study. Please see COREQ (COnsolidated criteria for REporting Qualitative research) Checklist.	Thank you for this comment. We have completed the COREQ checklist and hope that our methodology is now clearer.	
Abbreviations: Some need better clarification<br><br>People with (lived experience of) ADHD. Suggest you exclude lived experience. YP meaning what? Which are the ADHD supporters? What is YP?	Thank you. YP originally meant young people, but we have changed to P for person with ADHD for clarity.	Methods, Page 6<br><br>six people with ADHD (P)
Problem and background: I consider well written and easy to grasp.	Thank you for this comment.	
Background: Page 4, 47: ... a greater exposure to a range of health risks... in comparison to?	Thank you for this comment. This is in comparison to the general population, which we have now clarified.	Background, Page 4<br><br>"greater risk of certain health conditions compared to the general population"

4, 50-54... in comparison to) Reference 5-8 is not sufficient enough.	Thank you for this comment. We have cited more evidence towards this point.	
Scoping review (14) There is no reference in the text, and in the References is given BMJ Open 13:e068184, meaning?	Thank you for this comment. We are currently waiting for our scoping review to be accepted, and therefore we have put the citation as Under Review.	Background, Page 5<br><br>"Our previous scoping review on psychosocial interventions highlighted the need for a fuller conceptualisation of health and care needs in ADHD, as well as an increased availability and evaluation of interventions (Ward et al, under review)"
Aim: Adequate. However, the Purpose/aim is differently formulated in the manuscript. It is important to use the same formulation to avoid that something new is excluded or included.	Thank you for this comment. We have updated the objective in our abstract to provide better unity.	Objectives: This study aimed to explore the perspectives of people with ADHD, their supporters, and primary care professionals, on the wider physical and mental health needs of people with ADHD, and the support currently available.
Moreover, I advise you to use just "experience" or "personal experience" instead of "lived experience" since the latter is associated to specific qualitative approaches/methods.	Thank you for this comment. Whilst we accept your point, but in the interest of unifying publications across the MAP study, we would prefer to stick to the term 'lived experience'. We will change if you feel strongly about this, but we hope you can understand our perspective here.	
Following aspects (see below) should be clarified in detail to strengthen the trustworthiness (see Lincoln & Guba (1985) of the study and possibility to replicate the study.	Thank you; please see our comments below	
Design: A qualitative approach/method.... Clarify your theoretical assumptions and positioning. Inductive/ deductive approach? Manifest/latent level of	Thank you for this comment. We have clarified our use of inductive approach and that we approached data with a critical realist framework.	Methods page 9<br><br>For this project, we used reflexive thematic analysis, using framework methods in the manner described in Byrne and taking a critical

interpretation? References are needed.		realist approach [20,21] This was undertaken between July and August 2023. For details see the MAP study protocol [19].<br><br>In brief, a preliminary framework was developed using an inductive approach to the data, and assessing information power as analysis progressed [22]. This involved from line-by-line coding of initial transcripts by researchers (AP, RG, JW, KB)
Participants: Type of sample, recruiting procedure in detail, numbers of each participant group separately. Primary care professionals (number of GPs, number of Practice managers, number of Allied health care professionals (such as?), number of Persons/indiviuals/patients with individual/personal experience (or preferably, just use experience of) and number of supporters/carers. A clarifying table over the demographics of the participants is needed.	Thank you for this comment. As seen in our above responses, we have included greater detail on our type of sample and recruitment procedure. We provide detail in relation to numbers in each participant group, on. Page 7, methods.	Methods (Page 6/7)<br><br>“Four of these practices were urban, and one was rural. Three of these practices were in areas of high deprivation, and one of these practices served a large student population.”<br><br>“In total, 20 participants were recruited, consisting of five general practitioners (GPs), five practice managers (PMs), one mental health worker (MHW), six people with ADHD (P) and three supporters of people with ADHD (S). Demographics of the participants are given in Table 1.”
Data collection: How the data was collected is unclear. More details about the interviews are needed. Specify the type of interviews, number of interviews, the number of participants, and the	Thank you for this comment. We hope that we have provided sufficient clarity to your questions,	Methods, page 8:<br><br>Interviews were semi-structured and approximately one hour in length, and each participant only participated in one interview. Apart from one interview, with two

composition of participants per interview.		professional participants, all participants partook in one-to-one interviews with a researcher.
Please provide a table with the interview guides and the research questions.	Thank you. We have included the topic guide as a supplementary material.	Methods, Page 8.<br><br>"The questions relating to this paper are given in Table 2, whilst the full topic guide is available in supplementary materials."
Time of the data collection?	Thank you. We have provided this within the methods section.	Methods, Page 7:<br><br>Interviews took place between March and June 2023.
Analysis:<br><br>The given reference of the used method of analysis is confusing. The text describes the use of Byrne (2022) [15, 16] and (Braun and Clark and Gale et al, 2013). In the list of references Braun and Clarke (2006) is given. It is not sufficient to provide an article with how Byrne handled the RTA. What of the analysis processes have you used and how? What is the difference between the TA of Braun and Clarke (2006) and later Braun and Clarke RTA. Please clarify.	Thank you for this comment. We have now clarified this point in response to your above comment. We also reference the MAP study protocol, which provides more detail in relation to the analysis.	
Please provide a table over the analysis process to improve the transparency of the analysis. All the way from meaning unit (do not forget to include what interview, participant/number) to subthemes and themes.	Thank you for this comment. We would not usually provide a breakdown all the way to individual interviews as this risks a more reductionist approach. We would therefore leave this to Editor discretion.	

The writing "Reflective journals were kept aiding the process of coding and theme generation", in what way?	Thank you for this comment. We have provided more clarity around this point.	Methods, Page 9<br><br>"Reflective journals were kept aiding the process of coding and theme generation, and recognising the personal engagement with the research topic (e.g. clinical academics dealing with criticism of clinical services)."
Time of the analysis in relation to the data collection?	Thank you for this comment. We have clarified that this is between July and August 2023.	Methods, Page 8<br><br>"This was undertaken between July and August 2023.
I find the results difficult to grasp. The themes and subthemes seem adequate.<br>I recommend a short introduction /ingress for the reader which also describe how the quotes should be understood.<br>What interview? Is it a single participant or more? Patient or GP in what interview?	Thank you for this comment. We have provided a short ingress.	Results, Page 10<br><br>"From the 19 interviews conducted with 20 participants, three major themes were generated. These have been tabulated in Table 3."
The result consists of too many quotes. This way the result becomes too fragmented. What does YP and S mean? You need to clarify more in the quotes. In brackets you can insert a clarification of as for example "they" (patients) or "we" (GPs) for a better understanding of the quote. As a whole convert more of them to describing text.	Thank you for this comment. We have changed from abbreviations to fully written out roles for clarity. We have also provided clarity as to who is being referred to where indefinite articles are used.<br><br>In respect to converting more of the quotes to describing text, we do not feel that would add clarity to our manuscript, and we would rather allow readers to see the information we were presented with, especially given that this is an exploratory field. We feel that overall this is a question of style, and will accept guidance from the editor on this.	Results (Examples)<br><br>"She (Granddaughter with ADHD) went through.."<br><br>"pinning them (people with ADHD) down"
Table 1. The presentation of Themes and subthemes A subtheme in the table is	Thank you for this comment. We demoted peer support as a subtheme and forgot to update the table. We	See Table 3 (formerly table 1)

missing in the result section, Peer Dynamics. Theme description. "Their" refers to whom?	apologise for this oversight. We have removed the word there for clarity.	
Discussion<br><br>The discussion needs revision.<br>The discussion fails to appear when lifting aspects to discuss related to your own result and thereafter just write/refer to numbers in the list of references as (28,28, 39).	Thank you for this comment. We consider that the discussion integrates our results with the wider literature, and we have revised the discussion in this round of revisions. We hope that it is clearer but would appreciate any clarification on where revision is needed.	See various edits through discussion.
List of references<br><br>Please provide DOI when possible	Thank you. We will leave this to editor discretion.	
Contributions:<br><br>A reminder. In a qualitative study with inductive approach, you do not generate hypothesis.	Thank you for this comment. This is more in relation to the conception of the study (the hypotheses being those around our wider ADHD health work). We have changed to project conception to avoid the confusion you have pointed out.	

VERSION 2 – REVIEW

**REVIEWER NAME**	Poulton, Alison
**REVIEWER AFFILIATION**	University of Sydney, Sydney Medical School Nepean
**REVIEWER CONFLICT OF INTEREST**	Personal fees and non-financial support from Shire/Takeda, and book royalties from Disruptive Publishing (ADHD Made Simple).
**DATE REVIEW RETURNED**	11-May-2024

**GENERAL COMMENTS**	The authors have addressed the feedback of the reviewers and thoroughly revised and improved their paper. My only comment is that 'DNA' is still not interpreted in the participant quotation.

VERSION 2 – AUTHOR RESPONSE

Reviewer 2 Comments		
The authors have addressed the feedback of the reviewers and thoroughly revised and improved their paper. My only comment is that 'DNA' is still	We are glad that we have been able to sufficiently address your comments. We have now	“I don't know, a [did not attend] rate sometimes is really high for patients with ADHD, and it doesn’t matter how many times you invite them, they’ll still [did

not interpreted in the participant quotation.	corrected the omission of DNA. Thank you.	not attend] the next time." (Practice Manager)
Competing interests of Reviewer: Personal fees and nonfinancial support from Shire/Takeda, and book royalties from Disruptive Publishing (ADHD Made Simple).		
